# Recoverable, Record-High Lactic Acidosis in a Patient with Glycogen Storage Disease Type 1: A Mixed Type A and Type B Lactate Disorder

**DOI:** 10.1155/2016/4362743

**Published:** 2016-11-16

**Authors:** Yonatan Oster, Isaiah D. Wexler, Samuel N. Heyman, Elchanan Fried

**Affiliations:** ^1^Department of Medicine, Hadassah Hebrew University Hospital, Mt. Scopus, Jerusalem, Israel; ^2^Department of Pediatrics, Hadassah Hebrew University Hospital, Mt. Scopus, Jerusalem, Israel; ^3^Department of Critical Care Unit, Hadassah Hebrew University Hospital, Mt. Scopus, Jerusalem, Israel

## Abstract

A 17-year-old patient with GSD type 1a (von Gierke disease) was hospitalized with an extremely elevated serum lactate following an intercurrent infection and interruption of his frequent intake of carbohydrates. The patient developed shock, oliguric renal failure, and cardiorespiratory failure requiring mechanical ventilation and inotropes. At the peak of metabolic decompensation and clinical instability, serum lactate reached a level of 47.6 mmol/L which was accompanied by a severe anion gap metabolic acidosis with a pH of 6.8 and bicarbonate of 4 meq/L. The patient was stabilized with massive infusions of sodium bicarbonate (45 meq/h) and glucose and recovered without the need for dialysis. This patient illustrates pathophysiologic mechanisms involved in the development of extreme mixed type A and type B lactic acidemia, reflecting altered metabolic pathways in GSD type 1, combined with tissue hypoperfusion. The rationale for the specific interventions in this case is outlined.

## 1. Introduction

Lactic acidosis is an anion gap metabolic acidosis that results from lactate overproduction or underutilization. Based on the categorization of lactic acidosis developed by Cohen and Woods, the types of lactic acidosis can be classified as type A or B [1–3]. Type A lactic acidosis is usually secondary to a systemic process producing poor blood oxygenation or inadequate tissue perfusion resulting in overproduction of lactate (e.g., anaerobic muscular activity) or underutilization of lactate (e.g., hepatic insufficiency). Type B lactic acidosis is the result of lactate overproduction due to specific illnesses that interferes with intermediary metabolism including specific diseases such as diabetic ketoacidosis, specific drugs and toxins including metformin, and inborn errors of metabolism. Clinically lactic acidosis is associated with increased in-hospital mortality which can exceed 60% in patients with shock and lactate levels >4 mmol/L.

Glucose-6-phosphatase (G6Pase) deficiency (glycogen storage disease type 1, GSD1), also known as von Gierke disease, is one of the most severe forms of glycogen storage disease as lack of G6Pase deleteriously impacts on both gluconeogenesis and glycogenolysis. The inability to convert glucose-6-phosphate (G6P) to glucose causes severe postabsorptive hypoglycemia. Excess G6P is shunted to alternative pathways including lactate production [4, 5]. Metabolic homeostasis can only be maintained by frequent provision of exogenous carbohydrate which provides glucose for peripheral utilization and reduces flux through G6P producing pathways. Patients either with poor metabolic control or under stress which mobilizes G6P production can develop severe type B lactic acidosis. We report a case of a GSD1 patient with both poor metabolic control and a severely compromised clinical situation who developed extreme hyperlacticacidemia. This case illustrates the multiple pathways involved in the generation of this severe life-threatening condition and the challenges related to treatment that are not seen in other forms of lactic acidosis.

## 2. Case Report

A 17-year-old male patient diagnosed at infancy with GSD 1a, associated with the Arg83Cys in the G6Pase protein that is frequently found among Ashkenazic Jews, presented to the emergency room acutely ill. Since childhood the patient was fed with frequent carbohydrate rich meals during daytime and with a continuous corn-starch based formula at night administered through a percutaneous gastric feeding tube. The patient's level of metabolic control was suboptimal, and he suffered from severe hepatomegaly, nephromegaly, hyperlipidemia, and short stature. He had been hospitalized several times for severe lactic academia usually associated with fasting or nonadherence to the recommended diet. These episodes responded to treatment with dextrose and bicarbonate infusions.

The patient had been feeling ill for at least 7 days and had a 2-day history of nausea, weakness, and dyspnea. He had been unable to eat for several days and had not received his usual nighttime corn-starch based formula. Even though he had felt sick, he had delayed coming to the emergency room or notifying his physicians. Emergency medical personnel who were called to take him to the hospital noted respiratory distress and a blood glucose of 28 mg/dL. During transport, he was given intravenous dextrose.

On arrival to the emergency room, the patient appeared dyspneic with a Kussmaul respiratory pattern, pronounced tachycardia, and a state of high anxiety. His blood pressure was 143/78, and rectal temperature was 36.5°C. On physical examination, the patient was highly anxious and diaphoretic and had a firm enlarged liver. Laboratory testing done on admission indicated that the patient's hypoglycemia had been reversed (serum glucose of 133 mg/dL (7.4 mmol/L)), but there was a severe metabolic acidosis (pH of 7.11, HCO_3_, 4 mEq/L, and pCO_2_, 12.8 mmHg). The initial serum lactate was 27 mmol/L (normal range 0.5–2.2 mmol/L). Mild renal impairment (creatinine 79 *μ*mol/L) and mild leukocytosis were also noted.

**Figure 1:**
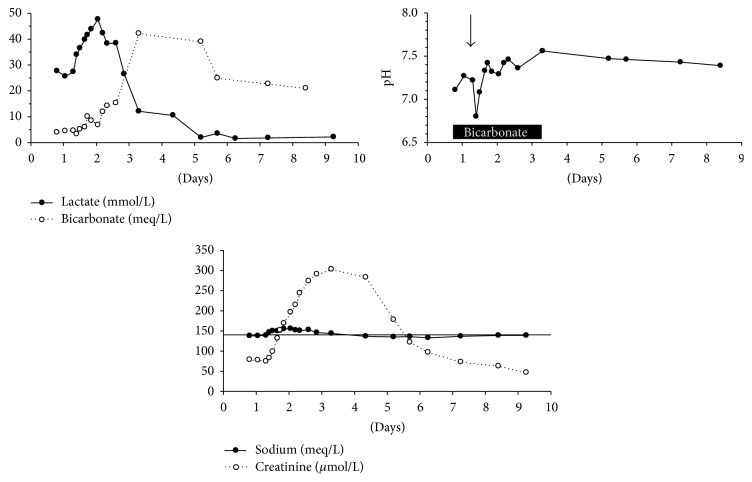
Changes in plasma pH and levels of lactate, bicarbonate, sodium, and creatinine during the hospitalization course. The black rectangle and arrow in the middle graph symbolize the period of bicarbonate infusion and the timing of initiation of mechanical ventilation, respectively. The horizontal line represents convenience sodium level of 140 meq/L.

The patient was initially treated immediately with intravenous dextrose, ceftriaxone, and sodium bicarbonate. Due to the worsening clinical situation despite treatment, endotracheal intubation and mechanical ventilation were required due to impending respiratory failure. Norepinephrine was administered for evolving hypotension and oliguria. Despite administration of bicarbonate at a rate of 45 mEq per hour, the metabolic status remained compromised with the blood pH falling to 6.8 and the lactate increasing to 47.6 mmol/liter (Figure 1). Additional complications during the course of treatment in the intensive care unit included hypernatremia (156 meq/L), related to bicarbonate administration and worsening azotemia (serum creatinine, 303 *μ*mol/L). At this stage, dialysis was considered as an option but was not initiated partly due to the reduction of lactate levels, beginning 26 hours after hospitalization. Recovery was progressive with support being gradually weaned. The patient recovered fully and discharged after 9 days of hospitalization with normalization of lactate, sodium, creatinine, blood pH, and serum bicarbonate. No precipitating cause was found for the metabolic crisis except for noncompliance with the dietary regimen; bacterial and viral studies were all negative, though during the course of the hospitalization, the patient developed* C. difficile* enterocolitis.

## 3. Discussion

We report a 17-year-old patient with GSD I who presents with extremely elevated serum lactate. In the past, the patient had suffered from metabolic crises related to his inborn error of metabolism, but the maximum lactate was milder and responded quickly when dextrose and bicarbonate were administered. In the current illness, even though serum glucose was normalized, lactate production was not diminished. What distinguished this incident from previous episodes of metabolic decompensation were the patient's associated circulatory shock, renal insufficiency, and severe catabolic state that lead to both overproduction of lactate and its underutilization.

**Figure 2:**
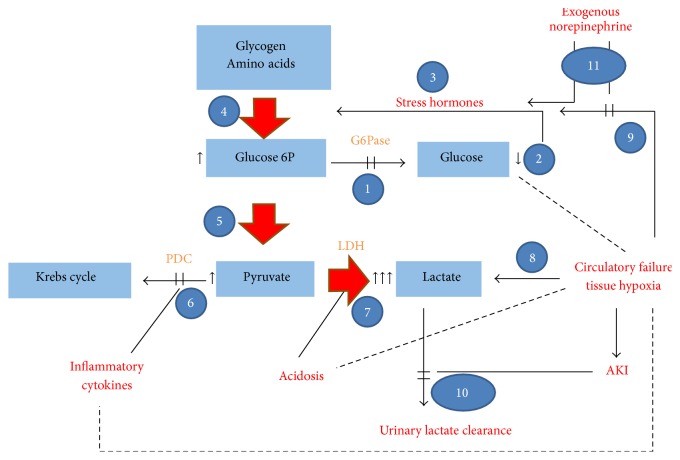
Suggested scheme of altered metabolic pathways and physiologic changes in the case report, leading to extreme hyperlactemia. The inherent disorder, nonfunctioning G6Pase, blocks conversion of G6P to glucose (1). In the absence of continuous glucose supplementation, ensuing hypoglycemia (2) activates stress hormones (catecholamines, glucocorticoids, and glucagon) and suppresses insulin secretion (3). Consequently, glycogenolysis and gluconeogenesis are enhanced, with accelerated generation of glucose-6P (4), which, unable to convert to glucose, undergoes glycolysis with the generation of pyruvate (5). Since PDC is inhibited by PDH kinase, induced by cytokines such as TNF and by depressed insulin, shunting pyruvate to the Krebs cycle is inhibited (6). Excess pyruvate is converted to lactate, especially in the presence of acidosis and with increased G6P (7). A type A component of lactic acidosis results from circulatory failure and tissue hypoperfusion and hypoxia, further intensifying lactate generation (8), and stimulating the release of stress hormones (9). Finally, urinary clearance of excess lactate is hampered due to evolving kidney failure (10). Exogenous catecholamines for circulatory failure (11) may reduce type A lactate generation by the restoration of tissue perfusion in some organs but might intensify type B lactic acidosis via enhanced gluconeogenesis. Scattered lines represent reciprocal association. LDH: lactate dehydrogenase; G6P: glucose-6-phosphate; G6Pase: glucose-6-phosphatase; PDC: pyruvate dehydrogenase complex; PDH: pyruvate dehydrogenase; AKI: acute kidney injury.

This case is an example of a combined type A and type B lactic acidosis. Type B lactic acidosis was caused primarily by the underlying metabolic disorder. As shown in Figure 2, there are multiple disrupted metabolic pathways in GSD 1 that lead to overproduction of lactate. In low glucose, low insulin states in healthy patients, G6P is produced primarily in the liver from both gluconeogenesis and glycogenolysis and then released into the blood stream as glucose after dephosphorylation by G6Pase. In the absence of G6Pase, G6P is shunted to other pathways including the glycolytic pathway. At the level of pyruvate, there are four pathways including (a) decarboxylation to acetyl-CoA, (b) carboxylation to oxaloacetate for utilization in gluconeogenesis and anaplerotic cycles, (c) transamination, and (d) conversion to lactic acid. In the low glucose, low insulin state, the pyruvate dehydrogenase complex is downregulated and pyruvate carboxylase is activated so as to generate more glucose. In patients with G6PD deficiency, increased flux in the gluconeogenic cycle causes futile cycling at the level of pyruvate kinase and recycling of G6P leading back to pyruvate. In the presence of high levels of pyruvate and NADH generated during overactivation of the glycolytic pathway, the lactate dehydrogenase reaction favors production of lactate. Gluconeogenesis conceivably was triggered by intrinsic stress responses, as well as by the administration of exogenous catecholamines.

The patient also had excess lactate production related to type A lactic acidosis that is associated with tissue hypoperfusion and enhanced anaerobic metabolism. The patient developed circulatory failure with shock, conceivably related to severe acidosis, necessitating the use of norepinephrine to support tissue perfusion. Tissue hypoperfusion, leading to anaerobic metabolism in muscle and other tissues, likely leads to increased peripheral production of lactate which would then be shuttled to the liver. The additional lactate load, even if taken up by the liver, could not be utilized for formation of glucose due to the metabolic defect and instead would be cycled back to pyruvate and subsequent reformation as lactate. Tissue hypoperfusion would also inhibit pyruvate oxidation via the pyruvate dehydrogenase complex in both the liver and peripheral tissues. This is especially true in the setup of acute inflammation, characteristic in critical illness in humans [6] and in experimental sepsis models [7] which is associated with enhanced expression of pyruvate dehydrogenase kinase 4 (PDK4), inactivating pyruvate dehydrogenase complex (PDC) [8]. Peripherally produced lactate uptake by the liver is reduced in the presence of acidosis and tissue hypoperfusion while the enzymes of gluconeogenesis are activated in the kidney by systemic acidosis.

Renal insufficiency likely also contributed to the extreme levels of lactate. With normally functioning kidneys, lactate excretion threshold is at plasma concentrations of about of 5-6 mmol/L, enabling removal of excess lactate. Chronic progressive renal failure, characteristic for patients with GSD 1, may lead to a priori defective lactate clearance and could facilitate lactate accumulation. Acute renal failure, as what happened in our patient, conceivably neutralized this safety valve mechanism, further contributing to the evolving record level of lactic academia.

The catabolic state resulting from the patient not eating for several days likely also led to protein breakdown and enhanced gluconeogenesis, intensifying pyruvate generation and transformation to lactate. Furthermore, it could lead to increased levels of acetyl CoA generated by fatty acid oxidation that would stimulate pyruvate carboxylation, generating additional precursors for the gluconeogenic pathway.

As a result of this unfortunate confluence of events, correcting hypoglycemia which is usually effective in children with metabolic crises in GSD 1 in this case did not stem the rising lactate. This patient was treated with massive amounts of sodium bicarbonate to preserve the buffering capacity of the extracellular space as the total loss of bicarbonate could cause a catastrophic lowering of blood pH. During the height of the metabolic crisis, dialysis was considered. Although bicarbonate-based hemofiltration has been recommended for severe hyperlacticacidemia and is utilized in metformin induced hyperlacticacidemia [9], it has been suggested that this would not be effective in the current situation as the rate of lactate clearance by dialysis seemingly is insufficient to offset the high rate of lactate generation specifically in type A lactic acidosis [10].

The patient's recovery was most likely due to the fact that the factors promoting type A lactic acidosis were reversed with fluid and supportive therapy which reduced both lactate production in the periphery and the stress reaction associated with critical illness. The type B lactic acidosis related to the underlying metabolic disorder was rectified by maintaining a normal blood glucose level and correcting nutritional deficits thereby increasing pyruvate oxidation, reducing gluconeogenic precursors, and inhibiting enzymes in the gluconeogenic pathway.

This case is instructive in showing that while type A and type B lactic acidosis are conceptually different, elements of both can coexist in the same patient. It is important to determine the etiology of lactic acidosis as the treatment for type A which is related to tissue hypoperfusion and enhanced anaerobic metabolism is different from type B in which the specific disease states, disorders of metabolism, or toxins cause either overproduction or underutilization of lactate. For the former, providing adequate tissue perfusion and oxygenation is the key intervention, while the latter requires treatment of the underlying disease.

## 4. Conclusions

The record level reversible lactic acidosis presented here reflects a unique combination of intense lactate generation and dysfunctional pathways involved in lactate clearance which are exacerbated in patients with G6Pase deficiency. With increasing life expectancy of such patients [11], reaching adulthood and even bearing children [12], it is anticipated that such outstanding cases may become prevalent with the need of critical care settings. Our report further illustrates that such extremely severe lactic academia can be successfully managed without dialysis. The fundamentals of therapeutic interventions consist of glucose replacement to attenuate lactate overproduction combined with vigorous efforts to correct acidosis and tissue hypoperfusion by judicious use of inotropes and fluid therapy.
